# A Scoping Review on Male-Mediated Developmental Toxicity

**DOI:** 10.3390/toxics13090707

**Published:** 2025-08-22

**Authors:** Lidia Caporossi, Paola Castellano, Enrico Paci, Daniela Pigini

**Affiliations:** Department of Occupational and Environmental Medicine, Epidemiology and Hygiene, INAIL, National Institute for Insurance against Accidents at Work, 00078 Monte Porzio Catone, Italy; p.castellano@inail.it (P.C.); e.paci@inail.it (E.P.); d.pigini@inail.it (D.P.)

**Keywords:** developmental toxicity, men, occupational exposure

## Abstract

Background: Developmental toxicity is defined as adverse effects induced either during pregnancy or as a result of parental exposure. While considerable attention has been devoted to maternal exposure to such chemicals, the role of paternal exposure has often been regarded as less significant. Objective: This study aims to highlight the impact of male-mediated developmental toxicity. Methods: An online search was conducted using PubMed, Scopus, and Google Scholar to identify studies focusing on developmental toxicity in offspring associated with paternal exposure during the preconception period. Results: The scientific literature—ranging from studies on pharmaceutical use to substances of abuse (notably tobacco, alcohol, opioids, and cannabinoids), as well as occupational and environmental exposure to specific compounds (e.g., phthalates, certain organic solvents, pesticides)—indicates that paternal exposure to developmental toxicants can adversely affect offspring health through various biochemical mechanisms. Conclusions: There is substantial experimental evidence of male-mediated developmental toxicity for various chemicals, demonstrating a particular vulnerability of the male germ line to transmissible effects. Several mechanisms have been proposed to explain the biochemical pathways underlying this toxicity. Evidence in humans is more challenging to interpret; however, numerous findings—both concerning substances of abuse and occupational exposures—raise concerns regarding the potential developmental risks to offspring.

## 1. Introduction

Sexual function and fertility in both adult males and females can be adversely affected by reproductive toxicity, as can the normal development of offspring through developmental toxicity.

The concept of developmental toxicity has been broadly defined in the Organization for Economic Cooperation and Development (OECD) draft Guidance Document on Reproductive Toxicity Testing and Assessment (GD 43) [1] as “…any effect that interferes with the normal development of the conceptus, either before or after birth, and resulting from exposure of either parent prior to conception, or exposure of the developing offspring during prenatal development, or postnatally, to the time of sexual maturation”.

The primary objective of classification under developmental toxicity is to provide hazard warnings to pregnant women, as well as to men and women of reproductive age.

Therefore, for practical classification purposes, developmental toxicity is essentially defined as adverse effects induced either during pregnancy or as a result of parental exposure. These effects may manifest at any stage of the organism’s lifespan. Developmental toxicity can lead to fetal death, structural abnormalities, impaired growth, and functional deficits [2].

For at least 70 years, researchers have considered the hypothesis that paternal exposure may influence offspring health [3]. Anthony Scialli [4] questioned whether the lack of unequivocal evidence of male-mediated developmental toxicity in humans reflects methodological limitations, rather than the absence of the phenomenon itself.

Pharmacological studies have contributed significantly to this debate, providing substantial evidence [5,6,7,8]. In particular, a large body of literature has addressed paternal-mediated toxicity associated with dermatological medications, including retinoids, immunosuppressants, antiandrogens, and thalidomide [6]. A review of 234 studies [5], covering 131 medications, identified 34 drugs associated with male infertility and sexual dysfunction, with 16 compounds directly implicated in concerns regarding teratogenicity. Some drugs, such as rosuvastatin [8], sevoflurane [9], and cyclophosphamide [10,11], are supported by experimental data demonstrating that paternal exposure can impair offspring health. Others, such as selective serotonin reuptake inhibitors (SSRIs), have shown certain adverse effects in humans, including increased risks of preterm birth [7], a mild increase in autism spectrum disorder risk in a cohort of 669 parents and 922 children [12], and a higher incidence of attention deficit hyperactivity disorder (ADHD) in offspring, as observed in a population of 781,470 subjects [13]. In all these studies, the fathers had to have taken the drugs at least 3 months before conception.

The question of how paternal exposure affects offspring health has become increasingly important, particularly given that men are generally subject to higher levels of potentially harmful occupational exposures compared to women [14]. Furthermore, the element of paternal age and the level of exposure can influence, even significantly, the toxic effects that can be recorded; in fact, higher male age is associated with a range of adverse health outcomes [15,16]. Public concern regarding environmental chemicals has been amplified by the growing understanding of epigenetic mechanisms at the molecular level [17,18,19], alongside an increasing number of studies addressing the multigenerational and transgenerational effects of environmental chemicals and lifestyle-related factors. To fully elucidate parent–child transmission effects, close collaboration between basic science and epidemiological research is essential [20].

In Europe, Directive (EU) 2022/431 [21] has aligned workplace risk management measures for substances toxic for reproduction with those established for carcinogenic and mutagenic substances, adopting a more precautionary approach for both male and female workers. In light of current regulations, some companies are beginning to question whether workplace exposure to substances harmful solely to fetal development should be addressed more broadly, rather than focusing exclusively on the protection of pregnant women. The legislation makes no distinction regarding the prevention and protection measures to be implemented in the case of reproductive toxicity affecting the fetus, nor does it differentiate between male and female workers. In fact, the answer to this concern is already implicit in the definition of developmental toxicants, which, unfortunately, is still too often overlooked with respect to paternal exposure.

However, the biochemical mechanisms underlying male-mediated developmental toxicity remain to some extent to be clarified, as does the identification of the chemical substances most frequently implicated in this phenomenon according to the scientific literature. This scoping review was aimed at mapping scientific evidence available in this field in a systematic way. The formulated research question was: What does the scientific literature report regarding paternal exposure to developmental toxicants during the preconception period and the potential adverse effects on offspring?

## 2. Methods

The present scoping review was conducted following the Preferred Reporting Items for Systematic Reviews and Meta-Analyses extension for Scoping Reviews (PRISMA-ScR) checklist [22]. The PRISMA checklist for scoping review is available as a Supplementary File.

### 2.1. Information Sources and Literature Search

A bibliographic search was performed from June to July 2025 using the Scopus, PubMed, and Google Scholar databases, employing the keywords “paternal exposure” and “developmental toxicity” or “male exposure” and “fetal toxicity.”

### 2.2. Inclusion Criteria

Articles, papers, books, and reports were included if they evaluated, compared, used, or described a situation of chemical exposure of men and related effects on offsprings. The types of chemicals had to be identified and the effects on the fetus or child had to be described.

Studies published in languages other than English, Italian, French, or Spanish were excluded. Articles documenting developmental toxicity solely in the context of maternal exposure were also excluded. Only studies investigating paternal preconception exposure leading to adverse developmental outcomes in offspring were included. In cases where both maternal and paternal exposures were assessed, the studies were excluded to avoid possible confounding factors. Grey literature was not considered. Surveys produced over the last thirty years have been collected.

The scoping review is concentrated on epidemiological investigations in human populations. Investigations focused on the effects due to the taking of drugs by fathers were excluded, as the type of “exposure” is linked to clinical needs, so considerations regarding the risk–benefit balance are the responsibility of the treating doctor. Instead, this scoping review wanted to focus on investigations in which exposure to chemical substances is essentially linked to work or specific living conditions. In particular, for investigations on occupational exposures, cohort studies were collected in which groups of those exposed and non-exposed were compared, or case/control investigations, in which the recruitment was conducted starting from the diagnoses of children, and therefore, the professional exposure was characterized retrospectively (also through a job exposure matrix).

### 2.3. Screening Process

The screening and data extraction processes of 4 reviewers were refined as they screened the same set of publications and discussed the results. The titles, abstracts, and full texts of the identified studies were rated sequentially by four researchers who worked in pairs. Discussion led to a consensus that resolved any disagreements about study selection and data collection, with additional reviewers if necessary.

A data-charting form was collaboratively developed by the four reviewers to define the variables to be extracted. Each researcher independently charted the data, after which the results were discussed, and the data-charting form was continuously updated.

Data abstracted included paper characteristics, with particular focus on the type of chemical exposure (occupational, environmental, substance abuse, or lifestyle-related). Experimental animal studies were included solely to illustrate proposed biochemical mechanisms.

**Figure 1:**
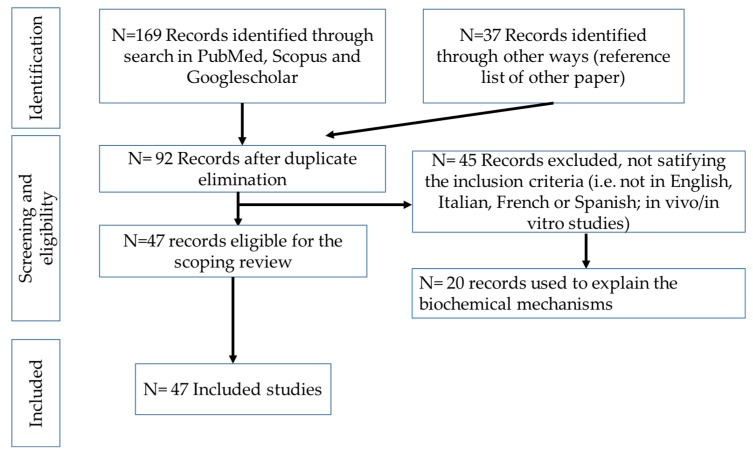
Selection process for scientific papers.

A schematic overview of the search strategy and the selected articles is presented in Figure 1.

## 3. Possible Biological Mechanisms

In vitro studies have already suggested that paternal exposure plays a significant role in fetal development. For example, an investigation into exposure to di-n-butyl phthalate [23] demonstrated that paternal exposure resulted in delayed sexual maturation in female offspring and a decline in seminal fluid quality in the male F1 generation. Similarly, studies on lead exposure [24] not only reported a reduction in seminal fluid quality in male rats but also observed a significant increase in pregnancy loss in females mated with these males.

There are multiple mechanisms through which paternal exposure to chemicals can influence conception outcomes [25,26].

1. Exposure to toxic contaminants in seminal fluid may directly affect the ovum or embryo. The blood–testis barrier is broken by many industrial chemicals, which can be detected in seminal plasma [27]. However, the real significance of this pathway remains unclear. In vitro fertilization assays could be useful to understand this pathway of toxicity; for instance, some studies [28] have reported that paternal exposure to certain chemicals, such as flame retardants, is associated with impaired fertilization of the oocyte.
2. Direct DNA damage in the germ line can be caused by chemicals, particularly affecting spermatogonia (point mutations), spermatocytes (aneuploidy), and spermatids (DNA strand breaks and chromosomal aberrations) [29]. Considering that a sperm cell’s ability to repair DNA fades during the last stage of spermatogenesis, it is not surprising that these effects have been demonstrated for many chemicals [26].
3. Development in subsequent generations may be directly or indirectly affected by paternal chemical exposure through interference with gene expression via imprinting and disruption of the epigenome. A proposed molecular mechanism involves DNA methylation, typically occurring at cytosine residues adjacent to guanine (CpG sites) [30], which can lead to the silencing of gene transcription in specific genomic regions. Another suggested mechanism is histone modification [17,31]. Although the replacement of most histones by smaller protamines occurs during spermatogenesis, approximately 5 to 10% of human histones persist in the sperm nucleus and remain unaltered [32,33]. Consequently, these histone modifications can be transmitted through spermatozoa. For example, paternal exposure to valproic acid in mice has been shown to induce behavioral deficits such as decreased social interaction, impaired pre-pulse inhibition, and non-spatial memory deficits. These effects have been linked to altered acetylation of histone H3 in the prefrontal cortex and hippocampus of the offspring [34].
4. Noncoding RNAs in seminal plasma are also suspected to play a role in transmitting paternal epigenetic information to offspring. Several small noncoding RNAs, as well as a limited number of mRNAs, have been shown to persist in sperm and enter the oocyte upon fertilization [35,36,37].

Offspring may exhibit a variety of intergenerational and transgenerational inherited effects resulting from exposure to numerous chemicals, including both bio-persistent and rapidly metabolized endocrine-disrupting compounds [18,19,38].

Environmental factors can impact offspring health through the paternal germ line, as supported by an increasing body of experimental evidence [39], although further biochemical research is necessary to fully elucidate the underlying pathways.

## 4. Evidence About Substances of Abuse: Alcohol, Tobacco Smoke, Opioids, Cannabis

The earliest and most evident effects reported in the scientific literature regarding male-mediated developmental toxicity involve substances of abuse, particularly alcohol, tobacco, opioids, and cannabis [40]. In these cases, the exposure levels and routes were well characterized, enabling a clear correlation to be established between paternal substance abuse and adverse developmental outcomes in offspring.

Numerous studies have demonstrated that substances of abuse can impair spermatogenesis and disrupt sexual hormone secretion via the hypothalamic–pituitary axis, thereby affecting sexual function [41,42,43]. Further proof shows that if paternal exposure to these substances occurs during preconception (at least three months before conception), it can lead to negative outcomes for the offspring. A summary of the effects associated with major substances of abuse is presented in Table 1. Specifically, paternal preconception exposure to tobacco smoke, alcohol, opioids, or cannabis smoke has been linked to neurodevelopmental impairments in offspring, including reduced mental health, hyperactivity, depression, attention deficits, and attention deficit hyperactivity disorder (ADHD) [44,45], likely mediated by epigenetic mechanisms [46,47,48,49,50].

Increased morbidity in offspring may manifest in both the short term (neonatal period and infancy) and long term (from childhood through adulthood).

## 5. Occupational and Environmental Exposure

Since the early 2000s, evidence has emerged indicating a decline in male seminal fluid quality, particularly in more polluted geographic regions, leading to the hypothesis that exposure to environmental pollutants adversely affects male reproductive health [76]. These areas have also reported higher incidences of stillbirth, certain fetal malformations, and spontaneous abortions, although a definitive causal correlation remains a subject of debate [77].

Conversely, studies conducted in occupational settings provide stronger evidence and clearer associations. An early systematic review from 1994 [78] classified the risk of spontaneous abortions or birth defects associated with male occupational exposure as “strong” for mercury and anesthetic gases, “moderate” for lead and organic solvents, and “limited” for pesticides. Subsequently, more methodologically robust studies have investigated solvent exposure in the painting industry, reporting increased risks of birth defects (OR = 1.86, 95% CI 1.4–2.5 [78]; and OR = 6.2, 95% CI 1.4–28 [79]).

A meta-analysis [80] highlighted how paternal exposure to solvents can be linked to a greater onset of neural tube defects (OR = 1.86, 95% CI 1.40–2.46) and anencephaly (OR = 2.18, 95% CI 1.52–3.11), while a weak correlation (RR = 1.19, 95% CI 1.00–1.41) between paternal exposure to some pesticides and the risk of hypospadias emerged. For spina bifida and solvent exposure, even with an OR higher than 1, the significance was lacking (OR = 1.59, 95% CI 0.99–2.56).

In contrast, another study [81] investigating the relationship between paternal pesticide exposure and cryptorchidism in offspring found no statistically significant association (OR = 1.04, 95% CI 0.96–1.12).

Positive associations were also reported between paternal occupational exposure to phthalates and polychlorinated compounds and the occurrence of congenital heart defects (OR = 2.08, 95% CI 1.27–3.40). Notably, specific subtypes of congenital heart defects showed stronger correlations: exposure to phthalates was linked to an increased risk of perimembranous ventricular septal defect (OR = 2.84, 95% CI 1.37–5.92), exposure to bisphenols with atrioventricular septal defects (OR = 4.22, 95% CI 1.23–14.42), and paternal exposure to alkylphenols was associated with coarctation of the aorta (OR = 3.85, 95% CI 1.17–12.67) [82].

A large cohort study [83], conducted between 1952 and 1988, enrolled 19,675 children born to 9512 fathers to assess the impact of paternal occupational exposure to chlorophenols—used as wood preservatives—on fetal malformations. Offspring of sawmill workers were the most affected by congenital malformations. Specifically, paternal exposure during the three months prior to conception was associated with an increased risk of eye malformations (OR = 2.87, 95% CI 1.5–5.5) and cataracts (OR = 5.68, 95% CI 1.4–22.6). No statistically significant associations were observed for undescended testes (OR = 1.16, 95% CI 0.8–1.6), genital malformations in general (OR = 1.29, 95% CI 0.9–1.5), or spina bifida (OR = 1.32, 95% CI 0.2–2.1). Another study [83] reported an increased risk of cleft lip in offspring of male farmers exposed to pesticides (OR = 3.00, 95% CI 1.03–8.70). However, the small sample size (35 cases and 35 controls) limits the strength of this finding.

A summary of the literature regarding occupational paternal exposure and pregnancy outcomes is presented in Table 2.

## 6. Conclusions

In this scoping review, we identified 47 primary studies focusing on dissemination and implementation research related to male-mediated developmental toxicity, encompassing both daily habits and occupational or environmental exposures.

More than half of the investigations concern substances of abuse, in which the levels of exposure, in terms of concentration and frequency, are certainly high and therefore have made it possible over the years to highlight effects, also for the development of conceived children, with greater clarity. Tobacco smoke, opioids, cannabis, and alcohol are mainly involved in this group of substances. The duration of substance abuse could be an important parameter, from a toxicological point of view, that is not always collected by the investigated subjects, which we think could be a limitation of some studies and a need for its introduction into future research. Similarly, considerations regarding the father’s age should always be taken into account as a confounding factor in epidemiological investigations.

On the other hand, the investigations conducted on working populations have focused attention on specific contexts (the production and use of paints, treatment of wooden materials, plastic industry) in which the use of specific chemical products is probably quantitatively consistent.

Our findings indicate that, particularly for certain chemicals, substantial evidence exists linking paternal preconception exposure to adverse effects in offspring.

There is robust experimental evidence demonstrating male-mediated developmental toxicity for numerous chemicals, highlighting a particular sensitivity of the male germ line to potentially transmissible effects. Several mechanisms have been proposed that may clarify the underlying biochemical pathways of toxicity. From a risk assessment perspective, it will be important for future research to focus on identifying a dose–response relationship, in order to allow for appropriate risk prevention measures.

Although human data are often complex and difficult to interpret, current scientific findings raise concerns about potential risks to offspring development arising from both paternal drug abuse (e.g., narcotics) and occupational chemical exposures.

When discussing reproductive health and embryonic development, the focus is often primarily on the mother. However, in recent years, increasing attention has been directed towards the paternal role, particularly concerning environmental and occupational exposures that can impact offspring health even before conception.

Unfortunately, too often, the protection of workers potentially exposed to endocrine disruptors has been, and still is, limited to women and strictly to the pregnancy period, without considering the long-term effects on fertility and possible genetic damage to and epigenetic effects on future pregnancies. Moreover, little attention is paid to the effects on men and the role that potential exposure to toxic or reprotoxic substances could have on future conception.

Organic solvents, pesticides, and substances such as alcohol, tobacco, and drugs are known to disrupt spermatogenesis and alter epigenetic mechanisms. While these exposures may not produce overt symptoms in adult men, they can induce lasting changes in the germ cells that give rise to sperm. Genetic or epigenetic modifications at this level can be transmitted to offspring, potentially influencing their development, metabolism, fertility, and susceptibility to diseases in adulthood.

Our scoping review has several limitations. First, studies addressing male-mediated developmental toxicity using in vitro and in vivo approaches were excluded by design. While this was an intentional eligibility criterion, it may have resulted in the omission of valuable information on certain chemicals. Second, the exclusion of publications not written in English, Italian, Spanish, or French limits our ability to capture evidence from studies published in other languages, potentially introducing a geographical bias. Lastly, we did not register our scoping review protocol. Although we do not view this oversight as a study limitation, per se, protocol registration is considered as best practice [86].

It is essential to develop prevention policies that recognize this risk and adopt appropriate preventive and protective measures, including the use of personal protective equipment, environmental controls, worker training, and, where possible, substitution of hazardous substances. As reported in the previous paragraphs, paternal exposure to chemical substances can significantly impact the health of the unborn child, underscoring the crucial role of the father—particularly in relation to environmental and toxicological factors that affect spermatogenesis and germline epigenetics. Therefore, it is essential to educate and inform prospective fathers about the potential risks associated with paternal exposure and its effects on offspring, especially since male-focused prevention is often overlooked in educational, healthcare, and informational programs. Preconception preventive efforts not only enhance reproductive health but also contribute to reducing the risk of chronic diseases in future generations.

Therefore, given the importance placed by the new Directive 2022/431 on protection from reprotoxic substances, it is advisable that occupational regulations extend protection to both sexes and, for women, not limit it solely to the pregnancy period.

## Figures and Tables

**Table 1:** Effects of substances of abuse on male reproductive health and offspring.

Type of Substance Use	Target of Reprotoxicity	In Detail	Effects on Offspring
Alcohol	Alters male reproductive hormones [41,51,52] Alters semen parameters [42,53,54] Testicular volume [55,56] Erectile and sexual function [57]	Higher: LH ^1^, FSH ^2^, DNA ^3^ fragmentation; lower: testosterone, seminal volume and sperm count, motility and morphology	Increased intrauterine growth restriction Increased birth defects, decreased birthweight Increased risk of cancers (leukemia and brain tumors) [41,58,59,60,61,62]
Cannabis (in particular THC ^4^)	Alters male reproductive hormones, semen parameters, libido, erectile and sexual function [42,43]	Lower: LH and sperm count, motility and morphology; higher: sperm DNA fragmentation. Testosterone levels were found to be both higher than lower	Increased pregnancy loss, increased congenital cardiac anomalies Increased behavioral issues [43,63,64]
Opioids	Chronic opioid use was linked with an increased risk of androgen suppression [65,66]	Lower: GRH ^5^ secretion, testosterone, sperm motility and morphology; higher sperm DNA fragmentation	Decreased fetal weight Increased withdrawal-like behaviors Increased risk of opioid addiction, delayed learning, and impulsive behaviors [67,68,69,70,71]
Tobacco smoke (nicotine in particular)	Alters sexual hormones, testis, and sperm [72,73]	Higher: testosterone levels; lower: sperm count, motility and morphology. Both higher and lower LH and/or FSH	Increased pregnancy loss Increased testosterone levels in child < 1 years Decreased sperm count and increased risk of neurodivergent behavior in childhood and adolescent [74,75]

^1^ LH—Luteinizing hormone; ^2^ FSH—Follicle-Stimulating Hormone; ^3^ DNA—deoxyribonucleic acid; ^4^ THC—∆^9^-Tetrahydrocannabinol; ^5^ GRH—Growth hormone-releasing hormone.

**Table 2:** Occupational exposure to chemicals of male workers and effects for offsprings.

Ref.	Chemicals	Effects for Offspring	Results
[81]	Solvents	Neural tube defects	OR ^1^ = 1.86, 95% CI ^2^ 1.40–2.46
	Solvents	Anencephaly	OR = 2.18, 95% CI 1.52–3.11
	Solvents	Spina bifida	OR = 1.59, 95% CI 0.99–2.56
	Pesticides	Hypospadias	RR = 1.19, 95% CI 1.00–1.41
[82]	Pesticides	Cryptorchidism	OR = 1.04, 95% CI 0.96–1.12
[83]	Phthalates and polychlorinated compounds	Congenital heart defects	OR = 2.08, 95% CI 1.27–3.40
	Phthalates	Perimembranous ventricular septal defect	OR = 2.84, 95% CI 1.37–5.92
	Bisphenols	Atrial ventricular septal defects	OR = 4.22, 95% CI 1.23–14.42
	Alkylphenols	Coarctation of aorta	OR = 3.85, 95% CI 1.17–12.67
[84]	Chlorophenate wood preservatives	Eye malformations	OR = 2.87, 95% CI 1.5–5.5
		Cataracts	OR = 5.68, 95% CI 1.4–22.6
		Undescended testicles	OR = 1.16, 95% CI 0.8–1.6
		Genital organs in general	OR = 1.29 95% CI 0.9–1.5
		Spina bifida	OR = 1.32 95% CI 0.2–2.1
[85]	Pesticides	Cleft lip	OR = 3.00 95% CI 1.03–8.70
[79]	Solvents in painting activity	Birth defects	OR = 1.86 95% CI 1.4–2.5
[80]	Solvents in painting activity	Birth defects	OR = 6.2 95% CI 1.4–28

^1^ OR—Odds ratio; ^2^ CI—confidence interval.

## Data Availability

No new data were created or analyzed in this study.

## Supplementary Materials

The following supporting information can be downloaded at: https://www.mdpi.com/article/10.3390/toxics13090707/s1, File S1: Reporting Items for Systematic reviews and Meta-Analyses extension for Scoping Reviews (PRISMA-ScR) Checklist.

## Abbreviations

The following abbreviations are used in this manuscript:

LH	Luteinizing hormone
FSH	Follicle-stimulating hormone;
DNA	Deoxyribonucleic acid
THC	∆^9^-Tetrahydrocannabinol; ^5^GRH- Growth hormone-releasing hormone
OR	Odds ratio
CI	Confidence interval
